# Preparation of controlled degradation of insulin-like growth factor 1/spider silk protein nanofibrous membrane and its effect on endothelial progenitor cell viability

**DOI:** 10.1080/21655979.2021.1982270

**Published:** 2021-10-20

**Authors:** Lifang Chen, Yulang Huang, Rongfeng Yang, Jian Xiao, Jiajia Gao, Debao Zhang, Duanwen Cao, Xiao Ke

**Affiliations:** a Department of Cardiology, Shenzhen Nanshan District Shekou People’ S Hospital, Shenzhen, China; b Department of Cardiology, Fuwai Hospital, Chinese Academy of Medical Sciences, Shenzhen Sun Yat-sen Cardiovascular Hospital, Shenzhen, China; c Clinical Trials Research Centre, The First Affiliated Hospital of Nanchang University, Nanchang China; d Key Laboratory of Biomaterials of Guangdong Higher Education Institutes, Department of Biomedical Engineering, Jinan University, Guangzhou, China

**Keywords:** Controlled-releasing, nanofibrous membrane, degradation, IGF-1, endothelial progenitor cells, TEV protease

## Abstract

The present study aimed to prepare a kind of controlled-releasing insulin-like growth factor 1 (IGF-1)/spider silk protein nanofibrous membrane using a electrostatic spinning method and evaluated its effect on the cell viability of endothelial progenitor cells (EPCs). Recombinant spidroin named as GMCDRSSP-IgF-1 was electro-spun into nanofibrous membrane which can be degraded by protease and be capable of sustained-release of IGF-1. The membrane can be degraded after being treated with thrombin. The release assay results showed that IGF-1 concentration could be maintained at 20 ng/ml for a long time with treatment of *Tobacco Etch Virus* (TEV) protease. The viability of EPCs on GMCDRSSP-IgF-1 nanofibrous membrane was significantly increased with the presence of TEV protease. The controlled and sustained release of IGF-1 from the nanofibrous membrane could promote the adhesion and viability of EPCs. In summary, the nanofibrous membrane that exhibits controlled degradation and sustained release of IGF-1 was prepared with electrostatic spinning from genetically modified recombinant spider silk protein. The nanofibrous membrane exhibited good blood compatibility and cytocompatibility. With the presence of TEV protease, the sustained-release of IGF-1 significantly promoted the adhesion and viability of EPCs. The new nanofibrous membrane can be potentially used as a scaffold for EPCs culture *in vitro* and future *in vivo* studies.

## Introduction

Cardiovascular diseases have caused more than 17 million deaths worldwide every year, accounting for 45.01% of deaths in rural area and 42.61% of deaths in urban area [1]. Atherosclerosis, hypertension, myocardial ischemia-reperfusion injury, and heart failure are common cardiovascular diseases or pathological processes [2–7]. However, their mechanism is not entirely clear. More and more studies showed that endothelial progenitor cells (EPCs) are not only one of the material sources of angiogenesis after birth, but also protect and repair blood vessels endodermis EPCs have stronger proliferation and differentiation potential, can differentiate into mature endothelial cells such as blood vessel cells, making it a hotspot in research of angiogenesis. EPCs have been shown to improve the early growth of new blood vessels in the body [8,9]. Studies have shown that vascular endothelial cell growth factor (VEGF), acidic fibroblast growth factor (aFGF), basic fibroblast growth factor, hepatocyte growth factor, platelet derived growth factor, stromal-derived factor-1 alpha, and insulin-like growth factors (IGFs) can regulate the proliferation, cellular activity, and physiological function of EPCs through different signaling pathways [10–12]. These cytokines have been suggested as necessary cofactors of EPCs in the treatment of cardiovascular diseases.

Insulin-like growth factor 1 (IGF-1) has a good application prospect in regulating the proliferation and physiological function of EPCs [12], but is easy to be degraded *in vitro* and *in vivo*, and has no sustained-release properties when administered alone [13,14]. Therefore, it is difficult to form a stable intracellular environment around EPCs, and the problem of short action time or excessively high local concentration is likely to occur. In recent years, the research on the controllable release of nanofibrous membrane in cell regeneration has attracted wide attention. It can not only provide scaffolds for cell growth [15] but also provide stable drug microenvironment for cells due to its capacity of drug sustained-release [12].

Studies have shown that membranes fabricated from spidroin are promising materials in biomedical applications [16]. It is feasible to apply them to a specific part of the human body as release materials for controlling substances, biomedical sensors, and cell support scaffolds [17]. Biomedical or biochemical sensors can be assembled onto recombinant spider silk protein by covalent binding of bioactive compounds [18]. It is also possible to combine substances (such as drugs) directly on the membrane, or to load these substances into particles and embed them in or add them to the laminar layer of the membrane for sustained release [19]. In addition, the membrane can be combined with specific functional groups through specific chemical or genetic means, and the hydrophilicity of the membrane surface can be changed to affect the adhesion, proliferation, and differentiation of cells on the membrane surface [20]. Membranes made from recombinant spider silk protein can also be used in medical applications, including coating medical devices [21] and skin grafts [22]. It is worth mentioning that the recombinant spider silk protein membrane can be partially degraded by wound hydrolase within 15 days, which is just the initial stage of wound healing [23]. Since the recombinant spidroin can be genetically modified, it is practical to insert a characteristic protease cleavage site into the recombinant gene to realize the controllable degradation of the recombinant spider silk protein.

In this study, we aimed to prepare a kind of controlled-releasing insulin-like growth factor 1 and nanofibrous membrane using a electrostatic spinning method and evaluated its effect on the cell viability of EPCs.

## Materials and methods

### Generation of genetically modified recombinant spidroins

Plasmids expressing the recombinant spider silk protein were obtained through gene synthesis, and then transformed into *E. coli* BL21 (DE3) cells [24,25]. Bacterial cells were inoculated in 10 ml LB medium containing ampicillin (100 mg/ml) and cultured in a shaking flask at 37°C to mid-log phase. Isopropyl-β-thiogalactoglucopyranide with a final concentration of 0.3 mM was added to induce the expression of the protein of interest. After incubation at 16°C and 180 rpm overnight, the cells were centrifuged at 6000 rpm for 15 min at 4°C, and then re-suspended in lysis buffer (20 mM Tris-HCl, 500 mM NaCl, pH 8.0). The cell suspension was then lysed using a high-pressure homogenizer (JNBIO, China). The cell lysate was centrifuged at 12,000 rpm for 30 min at 4°C to remove insoluble cell debris, and the supernatant was further loaded onto a non-denatured purification column filled with Ni-NTA sepharose (Qiagen, Germany). The protein solution and column were incubated on ice for 1 h, and then the column was washed three times with washing buffer (20 mM Tris-HCl, 500 mM NaCl, 10 mM imidazole, pH 8.0). Finally, eluent buffer (20 mM Tris-HCl, 500 mM NaCl, 250 mM imidazole, pH 8.0) was used to eluent the binding protein from the Ni-NTA column, and the protein eluent was transferred to dialysis bag and placed in buffer (20 mM Tris-Hcl, 500 mM NaCl, pH 8.0) for overnight dialysis at 4°C.

The ubiquitin-like-specific protease 1 (ULP1) protease was added in dialyzed protein solution with the volume ratio of 1:1000, then the mixture was incubated at room temperature for 1 h. The small ubiquitin-like modifier (SUMO) protein fragment was removed with the enzyme-cut mixture passing through Ni-NTA column three times. The solution flowing through the column was collected and transferred to a concentrated tube with a molecular interception of 14 kDa and centrifuged for 4000 rpm at 4°C until the concentration of the protein solution was 100 mg/ml [24,25].

### Preparation of nanofibrous membrane

**Figure 1.**
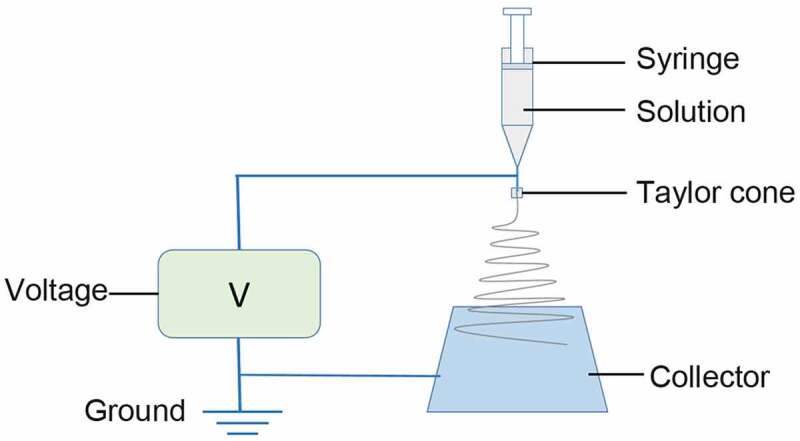
Schematic illustration of electro-spinning process

The nanofibrous membrane was prepared with an electro-spinning process (Figure 1) based on the previous study [26]. Briefly, the spinning dope was ejected with an LSP01-3A syringe pump (Longer Precision Pump Co. Ltd, Shanghai, China) through a 16 G syringe needle at the speed of 0.6 ml/h. The applied high voltage was set at 9 kV. A rectangle aluminum foil was used as a collector and positioned horizontally 14 cm from the needle tip perpendicularly and grounded. The collected nanofibrous membrane were cross-linked in ethanol vapor for 24 h and then dried for 24 h in a vacuum dryer. All the electro-spinning processes were carried out at around 25°C and 50% relative humidity.

### Scanning electronic microscopy of nanofibrous membrane

The morphology of nanofibrous membranes was examined with scanning electron microscope (SEM; Hitachi, Japan) at an acceleration voltage of 10 kV after sputter-coated with gold for 25 s [27]. The distribution of diameters of fibers was analyzed by Image J (National Institutes of Health, USA) from 100 randomly selected nanofibers on typical SEM images at a magnification of 10,000.

### Fourier-transform infrared spectroscopy (FTIR) analysis and thermogravimetric (TG) analysis

The chemical bonds and functional groups present in the nanofibrous membrane were analyzed from the FTIR spectra recorded with a Nicolet 6700 FITR spectrometer (Thermo Fisher Scientific, USA) [28] in the range of 4000–600 cm^−1^ in attenuated total reflection mode at room temperature. For each spectrum, 64 scans were averaged with a resolution of 1 cm^−1^. The standard thermogravimetric (TG) analysis was completed using a thermogravimetric analysis instrument (TA-SDT Q600, TA instruments, New Castle, DE, USA) (air atmosphere, heating rate: 5°C/min, 25–800°C) [29].

### Water contact angle measurement

Water contact angles on the membrane surface were measured with a drop shape analyzer DSA30 (KRUSS, Germany) with the sessile drop technique water contact angle [30]. All data were analyzed with ADVANCE-drop shape software to determine the contact angles. The final data were averaged from three measurements from three samples.

### Mechanical property testing

The mechanical properties of nanofibrous membrane were tested with a universal testing machine (H5K-S, Hounsfield, UK) with a crosshead speed of 10 mm/min under a load of 10 N [31]. All the nanofibrous membranes were cut into small strips with the width and gauge length of 10 mm × 30 mm, and three strips from different sites of each fibrous membrane sample were chosen for and the results averaged. The stress and strain data were calculated using Equations (1) and (2).

(1) $$\sigma \left(MPa\right)=\frac{P\left(N\right)}{w\left(mm\right)\times d\left(mm\right)}$$

(2) $$\varepsilon =\frac{l}{l_{0}}\times 100\%$$

where σ, ε, *e, P, w, d, l*, and *l*_0_ stand for stress, strain, load, sample width, sample length, extension length, and gauge length, respectively. Tensile strength, elongation at break, and Young’s modulus were obtained from the strain-stress curves.

### Circular dichroism (CD) spectrum analysis

CD spectra of spinning dope were recorded from 260 to 190 nm at 20°C using a Chirascan spectrometer (Applied Photophysics Ltd., England) in 300 μl quartz cuvettes with 0.1 cm path length, at a wavelength ramp rate of 0.5 nm, response time of 1 s, and bandwidth of 1 nm [32]. The proteins at concentration of 0.1–0.3 mg/ml. The sample was scanned three times and the spectra were averaged. Blank solutions were measured under the same conditions and subtracted from spectra. Software CDNN were used to estimate the percentage of different secondary structure from CD spectra.

### Wide-angle X-ray diffraction (WAXRD) analysis

WAXRD for nanofibrous membranes was performed on D/max-2550 PC X-Ray diffractometer (Japan Rigaku Corporation) [33] with Cu radiation (40kV /20 mA, λCu/Kα = 1.5406 Å) over the 2θ range of 5–60°.

### Controlled degradation of nanofibrous membrane and the sustained release of IGF-1

The nanofibrous membranes were cut into 10 mm × 30 mm strips. Samples were weighed and recorded as W_0_ after dried at 55°C for 24 h, then the samples were immersed in 10 ml phosphate buffer containing 1 U/ml thrombin protease (pH 7.4), sealed and placed in a 37°C shaker, and incubated at a shaking speed of 20 rpm. Samples were taken out every 48 h hand washed with a large amount of distilled water, dried, weighed and recorded as W_1_. The degradation rate is calculated using the following formula.

(3) $$Degradation\left(\%\right)=\left(w_{0}-w_{1}\right)/w_{0}\times 100\%$$

The nanofibrous membranes were cut into strips of 10 mm × 30 mm, then immersed in a 10 ml phosphate buffer (pH 7.4) containing 1 U/ml *Tobacco Etch Virus* (TEV) protease, sealed and placed in a 37°C shaker, and incubated at 20 rpm. 100 μl sample was taken out every 4 h and IGF-1 levels were determined by enzyme-linked immunosorbent assay and 100 μl phosphate buffered saline (PBS) was added.

### Hemolysis assays

Before the assays, the strips of nanofibrous membranes (10 mm × 30 mm) were immersed in 75% ethanol for 0.5 h for sterilization and washed three times with sterilized water. Healthy red blood cells (HRBCs) were obtained from fresh New Zealand white rabbit blood containing 3.8% sodium citrate injection (sodium citrate to distilled water ratio, 3.8:100 w/v) as anticoagulant (the ratio of anticoagulant to blood is 1:9, v/v). The assays were performed according to the previous literature [34]. In brief, the diluted HRBCs (0.2 ml) mixed with PBS solution (10 ml) with a total volume of 10.2 ml were added into centrifugal tube with bottom covered with nanofibrous membrane. The diluted HRBCs (0.2 ml) were mixed with 10 ml water as a positive control and 10 ml PBS buffer as a negative control for comparison, respectively. All samples were incubated at 37°C for 2 h. Samples were centrifuged at 2000 rpm for 5 min and the suspensions were taken away carefully. The absorbance at 545 nm of the supernatant (hemoglobin) was determined by Lambda 25 UV–Vis spectrophotometer (Perkin Elmer, USA). The hemolysis rate (HR) was defined as the following formula [35]:

(4) $$HR=\frac{SA-NA}{PA-NA}\times 100\%$$

where SA, PA, and NA stand for the absorbency of the experimental sample, the positive control and the negative control, respectively. Mean and standard deviations of the triplicate centrifugal tubes were calculated.

### Cell culture of EPCs

EPCs were obtained from Shanghai Institute of Biochemistry and Cell Biology (Shanghai, China). EPCs were seeded with a density of 1.0 × 10^4^ cells/well and cultured in Dulbecco’s modified Eagle’s medium (DMEM) with 10% fetal bovine serum and 1% antibiotic-antimycotic in an atmosphere of 5% CO_2_ and 37°C and the medium was replenished every 2 days.

### Cell seeding of EPCs on the nanofibrous membranes

Membrane samples were cut into discs of 2.5 cm in diameter and placed into 24-well plates individually and secured by stainless rings. Before cell seeding, the samples were sterilized by exposure to 400 mg/l ethylene oxide for 12 h. EPCs were seeded with a density of 1.0 × 10^4^ cells/well and cultured in DMEM with 10% fetal bovine serum and 1% antibiotic-antimycotic in an atmosphere of 5% CO_2_ and 37°C and the medium was replenished every 2 days.

### Cell counting kit-8 (CCK-8) assay

CCK-8 assay kit (Beyotime, Beijing, China) was sued to evaluate the viability of ECPs. The time points for CCK-8 analysis were set at 2, 4, and 7 days after seeding. At each time point, the culture medium was removed, and the samples were washed with PBS and the fresh medium was added into the wells. Then CCK-8 assays were performed according to the manufacturer’s protocol. The measurements were performed in triplicate.

### Statistical analysis

All results are represented as the mean ± standard deviation (SD). The data analyses were performed using GraphPad Prism software 6.0 (GraphPad Software, La Jolla, USA). Significant difference among different treatment groups was analyzed using one-way ANOVA followed by Bonferroni’s multiple comparison tests. P < 0.05 was considered statistically significant.

## Results

### Production of recombinant spidroins

In this study, through bioengineering technology, IGF-1 and gene sequences containing TEV protease site were inserted into the spider silk protein structure to construct a recombinant spider silk protein that can be degraded by TEV protease. In addition, IGF-1 was fused with the recombinant spider silk protein and can be released in a controlled manner by protease cleavage. The spider silk electrospun nanofibrous membrane was used as a scaffold to culture EPCs. The physicochemical properties of the membrane were studied by means of SEM, FTIR, WAXRD, and mechanical properties testing. The controlled degradation and sustained release of IGF-1 from nanofibrous membrane were systematically studied. The effects of the prepared nanofibrous membrane on the EPC viability and adhesion were also evaluated.

**Figure 2.**
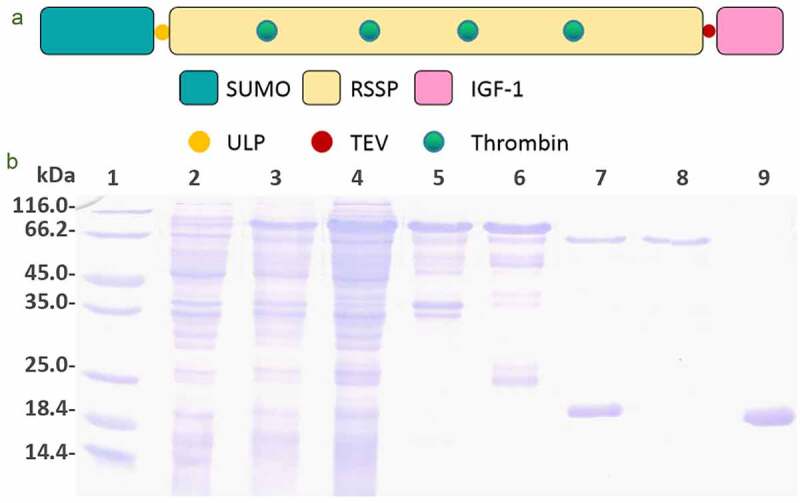
Protein production. (a) Schematic structure of SUMO-GMCDRSSP-IGF-1 spidroin. (b) The identification of the protein production. Lanes 1–9 are protein ladder, total protein before induction, total protein after induction, supernatant of cell lysate, precipitation of cell lysate, elution solution, mixture after ULP1 protease cleavage, GMCDRSSP-IGF-1 spidroin, and SUMO protein, respectively

The genetically modified controllable-degradable recombinant spidroin, which was named as SUMO-GMCDRSSP-IGF-1, composed of 755 amino residues with a theoretical molecular weight of 74.1 kDa. Its schematic structure is illustrated in Figure 2(a). In the N-terminus, there is an His-tag fused with SUMO peptide fragment (120 aa), which can promote the correct folding and dissolution of the newborn protein. The core part of the spidroins was composed of 565 amino residuals and was originated from spider silk gene. This part was inserted with five copies of thrombin enzyme digestion sequence (ENLYFQGLVPRGS) and was the trigger points for controlled degradation of silk peptide fragment. The C-terminus was fused with the IGF-1 sequence (70 aa) and a TEV protease digestion sequence on its N-terminus, which was designed for the sustained-release of IGF-1.

The plasmids expressing the SUMO-GMCDRSSP-IGF-1 spidroins were obtained by genetic synthesis and transformed into *E. coli* strain BL21(DE3). The protein production and purification were detected with Coomassie brilliant blue staining after SDS-PAGE electrophoresis. As shown in Figure 2(b), the interest protein was partially expressed as a soluble protein and partially expressed in the inclusion body. The His-tag and SUMO peptide fragment was cut off by ULP1 protease to get rid of the possible influence.

### Characterization of the nanofibrous membrane

**Figure 3.**
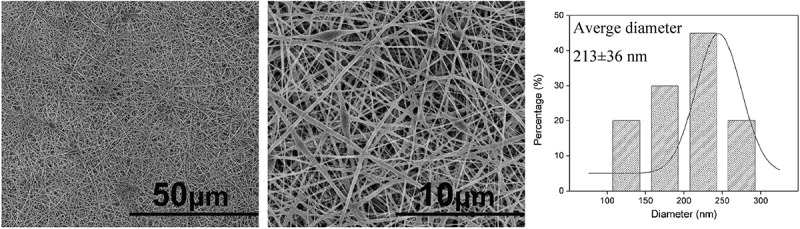
SEM images of nanofibrous membrane and diameter distribution histogram

The surface morphology and the diameter distributions of the membranes were observed by SEM (Figure 3). The membranes showed a framework of random nanofibers and displayed a porous three-dimensional structure. The surface of the nanofibers was smooth and the diameters of these nanofibers maintained a relatively narrow distribution range of 120–282 nm and the average diameter was 213 ± 36 nm.

**Figure 4.**
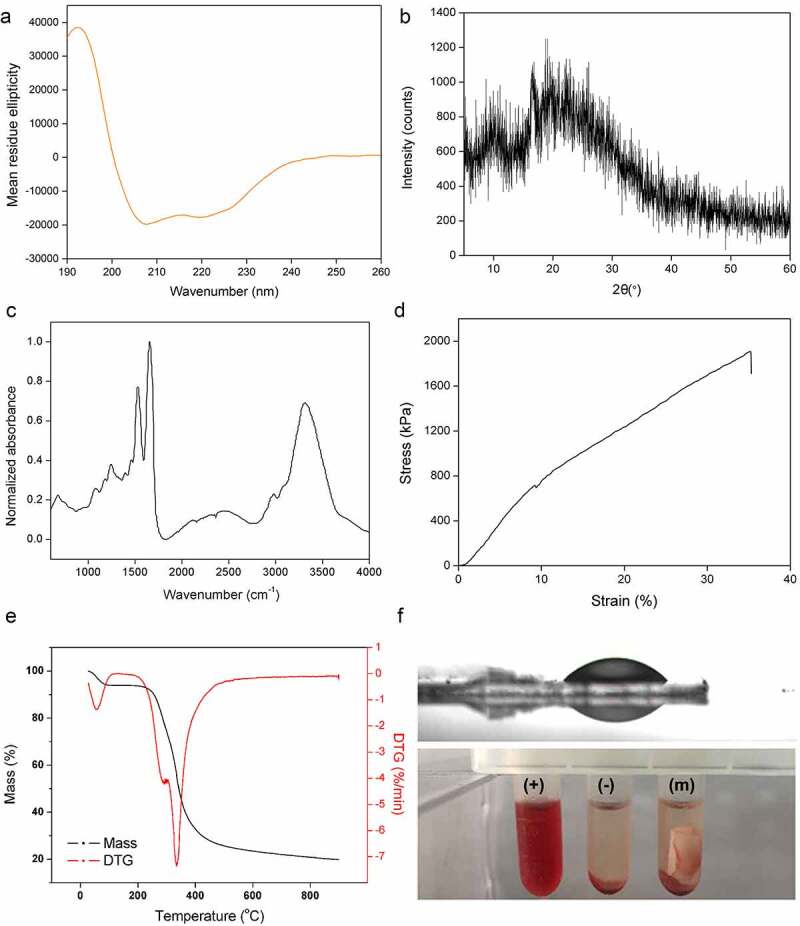
Characteristics of the nanofibrous membrane. (a) CD spectra. (b) WAXD pattern. (c) FITR spectra. (d) Typical stress-strain curve. (e) TG and DTG curves. (f) Water contact angle (up) and hemolysis assays (bottom). (+), (-) and (m) represent positive controls, negative controls, and nanofibrous membrane

The secondary structure of the spidroins in the spinning dope was estimated by analyzing CD spectra (Figure 4(a)). The results showed that the secondary structure of the GMCDRSSP-IGF-1 spidroins was dominated with α-helix and random coil which was in line with the previous reports of natural spidroins in spider silk glands [32].

The WAXRD pattern of the membrane exhibited some weak broaden Bragg peaks, which was due to Silk I and Silk II nanocrystal structures of recombinant spidroins (Figure 4(b)). For silk fibers, the micro-structure is nanocrystals embedded in amorphous matrix [36]. The results of weak and broad peaks suggested that main structures of this membrane are amorphous, indicating that the solution can be easily transported into the fibers.

In fibers, FTIR spectra showed strong peaks at 1640 cm^−1^, 1550 cm^−1^ and 3300 cm^−1^ which represented the stretching vibration of amide bond (amide I band), the presence of N-H (amide II band), and stretching vibration of N-H and hydrogen-bond interaction, respectively (Figure 4(c)). These main peaks indicated the amide I and II bands which were the key functional groups of protein. The analysis results of FTIR showed that the secondary structure contents in the fiber was quite different from spinning dope with decreased α-helix and increased β-sheet, indicating an α-helix to β-sheet transformation during spinning process, which was in line with the previous reports on the secondary structure changes during the wet-spinning process [37,38] and electro-spinning process [39]. For silk fibers, β-sheet structure provides the strength of mechanical performance.

The tensile strength of this membrane was around 1.9 MPa (Figure 4(d)) and the strain was over 30%. Figure 4(e) showed the TG and derivative thermogravimetric (DTG) curves of nanofibrous membrane. A slight decrease in weights on all curves below 100°C was due to the vaporization of water inside the nanofibrous membranes. The temperature of the thermal degradation onset was 260°C, which was caused by the thermal decomposition of the material. Meanwhile, when the weight dropped to 50%, the thermal temperature was ~350°C, and still ~20% of weight was retained at 900°C. From the DTG curve, it can be observed that the peak decomposition temperature of pure nanofibrous membrane reached at ~330°C. The results indicated that the membrane possessed the similar thermal stability to natural spider silk [40].

The hydrophilicity or wettability has been reported to have great influence on the initial adhesion and proliferation of cells [41]. A hydrophilic surface is conducive to absorbing protein then providing a benign condition for cell attachment, differentiation, and migration. The water droplets on the membrane surfaces in Figure 4(f) (up panel) showed hydrophilic surface with the water angle of 18 ± 0.7°.

Blood compatibility is one of the decisive factors in engineering scaffolds. Physical and chemical substances on the surface damage red blood cells, leading to the release of hemoglobin. The hemolysis test results (Figure 4(f), bottom panel) showed that the HR of nanofiber membrane was 0.3%, which was much lower than the 5% standard of international biomaterials.

### Controlled degradation of nanofibrous membrane

**Figure 5.**
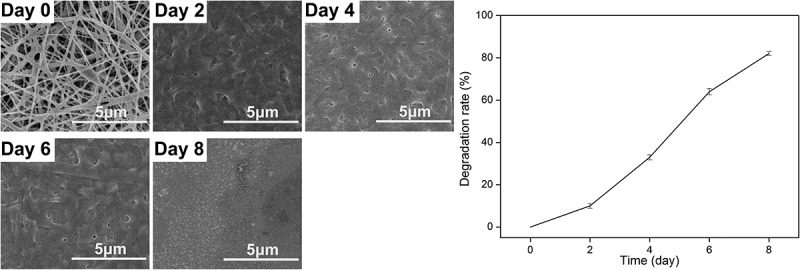
Degradation testing results of GMCDRSSP-IGF-1 spidroins *in vitro*. SEM images of degraded nanofibrous membrane (Left) and degradation curve (Right)

The results of controllable degradation *in vitro* are shown in Figure 5. After 1- or 2-day treatment with thrombin, the lost mass of membrane can be up to 10%. The membrane began to collapse after a 4-day treatment. More than 80% mass was lost after a 8-day treatment. The results showed that the GMCDRSSP-IGF-1 spidroins can be degraded in a controlled manner.

### Sustained release of IGF-1

**Figure 6.**
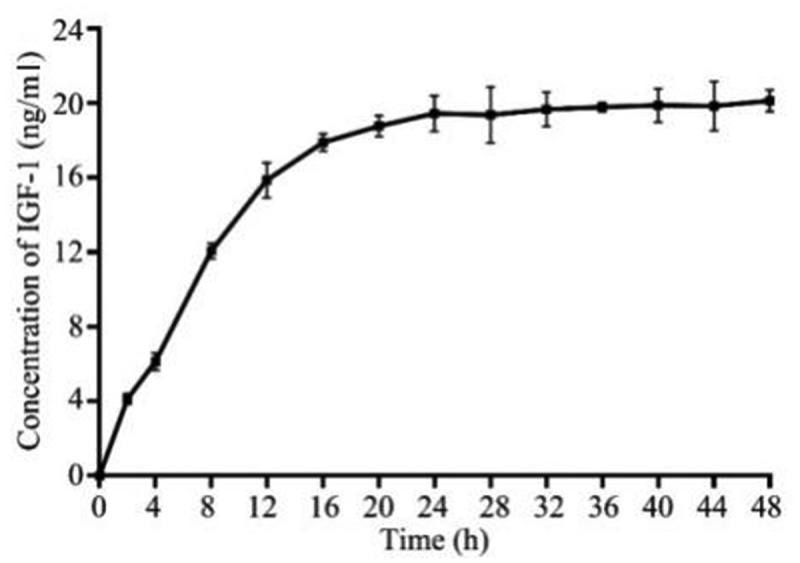
Controlled sustained-release performance test *in vitro* of GMCDRSSP-IGF1 spidroins

IGF-1 is prone to in degradation *in vivo* and *in vitro*, so it exhibits short-time application of a sudden release with high local concentration instead of sustained release. A cleavage site for TEV protease was designed between the main structure of spider silk protein and IGF-1 in GMCDRSSP-IGF-1 spidroins, endowing the membrane with a sustained-release performance. Results of sustained-release of IGF-1 *in vitro* are shown in Figure 6 demonstrated that GMCDRSSP-IGF-1 spidroins could sustained release IGF-1 with the presence of TEV protease. The highest concentration of IGF-1 can be maintained at a concentration of 20 ng/ml for up to 48 h.

### Viability of EPCs

**Figure 7.**
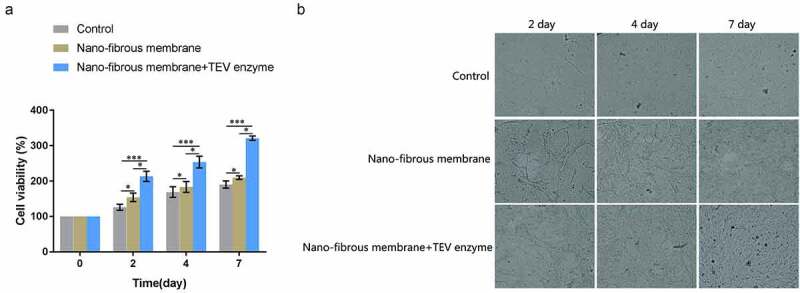
Effects on the proliferation of EPCs cultured on GMCDRSSP-IGF-1 nanofibrous membrane *in vitro.*

Finally, the effect of GMCDRSSP-IGF-1 spidroins nanofibrous membrane on the viability of EPCs was examined in the presence of TEV protease. The results showed that, compared with the blank control group, the viability of EPCs on the nanofibrous membrane group and the nanofibrous membrane + TEV protease group increased significantly after 4 and 7 days (Figure 7). Compared with the nanofibrous membrane group, the viability of EPCs in the nanofibrous membrane group +TEV enzyme group increased significantly after 4 and 7 days (Figure 7). The results showed that GMCDRSSP-IGF-1 spidroins nanofibrous membrane could significantly increase the viability of EPCs with addition of TEV enzymes.

## Discussion

Cardiovascular disease is a serious threat to the aged, especially those over 50 years, and is characterized by high morbidity and mortality. Even with the most advanced and perfect treatment, more than 50% of survivors fail to make a full recovery. Cell-based therapies by stimulating postpartum angiogenesis or repairing vascular integrity are being evaluated for cardiovascular diseases including ischemic heart disease, in-stent restenosis, pulmonary hypertension, and peripheral artery occlusion.

Cytokines, such as IGF-1, have a good application prospect in regulating the proliferation and physiological functions of EPCs, but they are easy to degrade *in vivo* and *in vitro*, and have no sustained-release properties during drug administration, and are prone to problems of short action time or excessively high local concentration. In this study, a novel recombinant spidroins, which was composed of IGF-1, spider silk protein, thrombin, and TEV enzyme cleavage sites were deliberately designed and obtained by ferment of low-cost bacteria. The electrospun nanofibrous membrane exhibited physicochemical properties which were suitable for cell culturing. Its hydrophilic surface is conducive for cell attachment and proliferation. It is strong enough to support cell growth. Low hemolysis rate told its safety of being used as biomaterials. The novelty of the nanofibrous membrane was the combination of controlled-degradation and sustained-release of IGF-1. As shown in the results of *in vitro* degradation experiment, the membrane collapsed after 4-day treatment and around four-fifths mass lost after 8-day treatment with the presence of thrombin enzyme. EPCs have been recognized as a special group of cells that play an important role in maintaining endovascular balance and the pathogenesis of various diseases. EPCs can improve all functions of ischemic tissues and reduce organ damage through neovascularization [42]. Autologous endothelial progenitor cell transplantation can promote vascular endothelial repair in mice and enhance endothelialization [43]. Due to the controllable sustained-release of IGF-1, EPCs can grow better on the membrane according to the cell culturing assays.

Spider silk has outstanding mechanical properties, such as high breaking energy and exceptional toughness, which makes it attractive for medical, military, and industrial applications [44]. Zhou et al., prepared a novel electrospun spider silk fibroin/poly (d,l-lactide) composite fiber, and the composite fibers showed excellent biocompatibility according to the results from the evaluation of cytotoxicity [44]. Zhao et al., prepared wound membranes of polyvinyl alcohol and recombinant spider silk protein by electrospinning, and experiments of applying electrospun membranes as wound dressing for Sprague Dawley rat wound healing showed that it could promote wound healing and basic fibroblast growth factor expression [44]. Park et al., fabricated an electrospun silk fibroin with macropores that can be altered using the salt-leaching method, and the results indicated that electrospun silk fibroin may be a good bone substitute for large bone defects because of its extracellular matrix-like structure, biocompatibility, and osteoconductivity [45]. Recently, Lian et al., prepared spider silk protein nanofibrous membrane dressings loaded with sodium hydrogen sulfide/EPCs, which could promote wound healing efficiency through hydrogen sulfide and EPCs [46]. Consistently, we successfully prepared the spider silk protein nanofibrous membranes loaded with IGF-1/EPCs, which significantly promoted the adhesion and viability of EPCs.

There are several limitations that should be considered regarding the present findings. First, the effects of IGF-1/spider silk protein nanofibrous membranes on the adhesion and viability of EPCs were examined at the preliminary stage, and further detailed mechanistic studies should be performed to explore the underlying molecular mechanisms. Second, the present study was limited to *in vitro* functional results, and further *in vivo* studies are warranted to explore the potential application of the nanofibrous membranes in the treatment of cardiovascular diseases. Third, the localization of the EPCs in the nanofibrous membranes has not been examined, which should consider in future studies.

## Conclusion

In summary, nanofibrous membrane exhibiting controlled-degradation and sustained-release of IGF-1 was prepared with electrostatic spinning from genetically modified recombinant spider silk protein. The nanofibrous membrane exhibited good blood compatibility and cytocompatibility. With the presence of TEV protease, the sustained-release of IGF-1 significantly promoted the adhesion and viability of EPCs. The new nanofibrous membrane can be used as a scaffold for EPCs culture *in vitro* and future *in vivo* studies.
